# Contribution of the Caspase Gene Sequence Diversification to the Specifically Antiviral Defense in Invertebrate

**DOI:** 10.1371/journal.pone.0024955

**Published:** 2011-09-19

**Authors:** Bin Zhi, Lei Wang, Guangyi Wang, Xiaobo Zhang

**Affiliations:** 1 Key Laboratory of Conservation Biology for Endangered Wildlife of the Ministry of Education, Key Laboratory of Animal Virology of Ministry of Agriculture and College of Life Sciences, Zhejiang University, Hangzhou, The People's Republic of China; 2 Key Laboratory of Marine Biogenetic Resources, Third Institute of Oceanography, State Oceanic Administration, Xiamen, The People's Republic of China; 3 Department of Oceanography, University of Hawaii at Manoa, Honolulu, Hawaii, United States of America; National Institute on Aging, United States of America

## Abstract

Vertebrates achieve adaptive immunity of all sorts against pathogens through the diversification of antibodies. However the mechanism of invertebrates' innate immune defense against various pathogens remains largely unknown. Our study used shrimp and white spot syndrome virus (WSSV) to show that *PjCaspase*, a caspase gene of shrimp that is crucial in apoptosis, possessed gene sequence diversity. At present, the role of gene sequence diversity in immunity has not been characterized. To address this issue, we compared the *PjCaspase* gene sequence diversities from WSSV-free and WSSV-resistant shrimp. The sequence analysis indicated that the *PjCaspase* gene from the WSSV-resistant shrimp contained a special fragment, designated as fragment 3 (221–229 aa). Down-regulation or overexpression of the *PjCaspase* gene containing fragment 3 led to significant inhibition or enhancement of virus-induced apoptosis, but had no effect on bacterium challenge. We found evidence that the silencing or overexpression of this gene led to a 7-fold increase or 11-fold decrease of WSSV copies, respectively. Our results suggested that the *PjCaspase* gene containing fragment 3 provided the molecular basis for the antiviral defense of shrimp. This study represented the first report of the role of gene sequence diversity in the immunity of an invertebrate against virus infection. Invertebrates may employ this gene sequence diversity as a system to avoid pathogen interference with their immune response.

## Introduction

The non-specific innate immunity of invertebrates, including their humoral defenses and their cellular defenses such as phagocytosis and apoptosis, is the sole mechanism for them to defend themselves against invading pathogens [1]–[3]. They achieve these defenses through reorganizing pathogen-associated molecular patterns of the host's cell-surface proteins [4], [5]. Recently, model systems such as *Drosophila melanogaster* and *Caenorhabditis elegans* have enabled researchers to learn much about this innate immunity [6]–[9]. Nevertheless, our understanding of the innate immunity of invertebrates against a variety of noxious microbial pathogens remains largely incomplete. Several lines of evidence indicate that invertebrates exercise some degree of specificity against microbial pathogens and that the specificity likely results from diversifying the sequence of their immune genes [10]–[13].

The sequence diversification of the somatic genes, which encode recognition molecules, effector-enhancement molecules and other immune molecules, has been postulated as contributing to the immune specificity of invertebrates [14]. The biological significance of gene sequence diversification in understanding the innate immunity of invertebrates is similar to the significance of diversifying the receptors of the adaptive immunity system in vertebrates. Furthermore, the pre-existing host-defense mutation mechanism is simply co-opted and enhanced in the novel context of obligatory combinatorial diversification [4]. Clearly, understanding the sequence diversification of immune genes in innate immunity may provide critical information about defending invertebrates against microbial pathogens.

Programmed cell death (i.e., apoptosis), as the major component of the cellular defense mechanism, is an essential immune response to protect invertebrates from pathogen infection [15]–[17]. When pathogen-infected cells undergo apoptosis, the pathogens duplicated in these cells are not able to diffuse and infect other cells. Thus, apoptosis can significantly improve the anti-pathogen response of invertebrates. Caspases, a family of structurally-related cysteine proteases, contain two different subgroups that play essential roles in apoptosis: initiator caspases and effector caspases [18].

The initiator caspases (caspase-8, -9 and -10) can be triggered by either intracellular or extracellular stimuli, and are activated by a complex course of autocatalytic processing or conformational change. The activated initiator caspases specifically activate effector caspases (caspase-3, -6 and -7) to initiate the apoptotic process, resulting in an intricate cascade of events including interactions among several families of proteins, i.e., caspases, Bcl-2 family proteins and inhibitors of apoptosis proteins. Among these proteins, caspase-8 is required for all death receptor–mediated apoptotic pathways; caspase-3 has been recognized as the crucial executioner caspase, influencing the impact of a given apoptotic stimulus.

The caspase gene *PjCaspase* of the shrimp *Marsupenaeus japonicus*, showing a sequence similarity to the caspase-8 of other species, is an initiator gene that is triggered by cell signals during the shrimp's hemocytic apoptosis to defend against virus infection [16]. The shrimp possesses an open circulatory system, in which the hemocytes (circulating cells) can be analogized functionally to vertebrate leukocytes [19]. Shrimp are economically-important marine invertebrate [20], but in recent years, white spot syndrome virus (WSSV) has become a major pathogen that is devastating the shrimp farming industry worldwide. The WSSV is an enveloped, large, double-stranded DNA virus with an obovate morphology and a single filamentous appendage. Elucidating shrimp's immunity to WSSV would aid greatly in finding how to treat shrimp against this disease.

In this study, our results indicated that the *PjCaspase* gene possessed sequence diversity. Analysis of the gene sequence diversities of the virus-free and the WSSV-resistant shrimp showed that the *PjCaspase* gene containing a specific sequence at position 661 bp–687 bp (referred as fragment 3). Fragment 3 was enriched in WSSV-resistant shrimp, modulating virus-induced apoptosis. Furthermore, the results of in vivo experiments showed that the *PjCaspase* gene containing fragment 3 was specifically responsible for the hemocytic apoptosis induced by the virus and specifically took effect on the innate immune response against virus infection. Overall, our results indicated that the *PjCaspase* gene sequence diversity protected the shrimp against WSSV infection. This study represented the first report that shrimp employed gene sequence diversification as immunity to defend against virus infection.

## Results

### Identification of the *PjCaspase* gene sequence diversification

**Figure 1:**
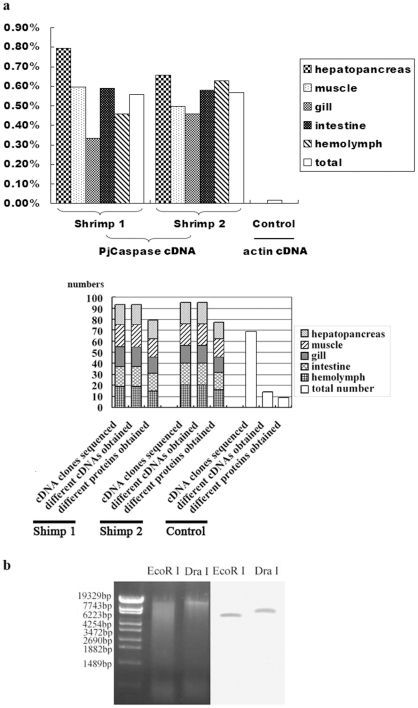
The sequence diversity of the shrimp *PjCaspase* gene. **a.** Frequencies of base mutations (up) and numbers of cDNA clones (down) sequenced for the *PjCaspase* gene of two virus-free shrimp (shrimp 1 and shrimp 2) and the control *β-actin* gene of shrimp presented at the nucleotide (nt) and amino acid (aa) levels. The organs or tissues analyzed were shown on the right. The cDNA clones of a tissue or organ were obtained from the same RNA template of a single shrimp. The GenBank accession numbers of the diversified gene sequences were GU645234∼GU645341 and GU645342∼GU645436. **b.** Southern blot of shrimp genomic DNA with a DIG-labeled *PjCaspase* probe that could detect all mutations of the *PjCaspase* gene. The genomic DNA extracted from shrimp hemolymph was digested with DraI or EcoRI and separated by electrophoresis on a 0.7% agarose gel (left). The blot was probed with a DIG-labeled *PjCaspase* gene (right).

Two classes of caspases, designated the initiators and the effectors, are the central components of the apoptotic response. *PjCaspase*, classified as the initiator of apoptosis, affects the immuno-possession in shrimp [16]. Our sequence analysis showed that the *PjCaspase* cDNAs that derived from the same pool of the same RNA template of a single shrimp presented a much higher frequency of somatic base mutations than the sequence analysis of the *actin* gene, a house-keeping gene, which we used for comparison (Fig. 1a, Figs. S1 and S2) (GenBank accession numbers GU645234∼GU645341). We found a higher frequency of base mutations of the *PjCaspase* gene in all the tissues and organs we sequenced. In total, the frequency of base mutation was 0.56% for the *PjCaspase* gene and 0.02% for the *actin* gene (Fig. 1a, up). When comparing the *PjCaspase* cDNAs from the same RNA template of an individual shrimp (Shrimp 1), we found that the *PjCaspase* cDNAs in a tissue or organ were completely different from each other due to base mutations, while most of the actin cDNAs were consistent (Fig. 1a, down). This phenomenon existed in all the tissues or organs we examined (Fig. 1a, down).

Further sequence analysis indicated that the somatic mutations in the *PjCaspase* cDNA sequences yielded no stop codons in the *PjCaspase* open reading frame (ORF), except for 3 cDNAs (Figs. S1 and S2). The data from control experiments excluded experimental artifacts caused by reverse transcription and PCR amplification (Fig. 1a). Therefore, our results suggested the existence of the cDNA sequence diversity of *PjCaspase* gene, a phenomenon widely revealed in invertebrates [21].

Cluster analyses using the partial sequences of *PjCaspase* cDNAs classified these cDNA sequences into 2 and 3 clusters at the nucleotide and amino acid levels, respectively (data not shown). The statistical analyses of these clusters indicated that the sequence diversification of *PjCaspase* cDNA in the shrimp was not organ- or tissue-specific. To explore the difference of gene sequence diversity between various individuals, the *PjCaspase* cDNAs from another individual shrimp were sequenced (Shrimp 2) (GenBank accession numbers GU645342∼GU645436) (Fig. S2). The cluster analyses yielded essentially similar results as those of Shrimp 1, showing no difference in the cDNA sequence diversity of the *PjCaspase* gene among these two individual shrimp. Because the sequences analyzed came from an individual shrimp, the sequence diversity revealed in this study should have been derived from somatic mutations, rather than from polymorphism among individuals.

The Southern blot analysis of the *PjCaspase* gene using the shrimp genomic DNA was conducted with a specific DIG-labeled probe (Stringency: 0.03 pg/µl) which could detect all the mutations of the PjCaspase gene. The results showed that the Southern blots yielded only one band (Fig. 1b), suggesting that the gene had a single copy in the shrimp genome. Thus, the sequence diversification did not result from the copy variation of the *PjCaspase* gene.

To determine whether the sequence diversification of the *PjCaspase* gene occurred in the genomic DNA, 44 clones containing the genomic DNA of the *PjCaspase* gene were sequenced (Fig. S3). The clones were obtained by PCR from the same pool of the DNA template of a single shrimp. The results showed that the sequences of the 44 genomic DNA clones of the *PjCaspase* gene were different from each other due to dot mutations (Fig. S3a). On the other hand, the genomic DNA sequences of the *actin* gene were identical (Fig. S3b). The frequency of base mutation was 1.58% for the *PjCaspase* gene and 0.01% for the *actin* gene. These results indicated that the sequence diversification of the *PjCaspase* gene occurred at the genomic DNA level.

### Effects of *PjCaspase* gene sequence diversity on virus-induced apoptosis

Gene sequence diversity has been reported in invertebrates [21]. However, the role of this diversity has not yet been addressed. The existence of gene sequence diversity of the key gene (*PjCaspase*) in antiviral apoptosis led to interest in its role in immunity. When the shrimp was invaded by various pathogens, would the products of the diversified *PjCaspase* genes play roles in the immune defenses to initiate the apoptosis against different pathogens? To address this issue, the diversities of the *PjCaspase* gene sequence from WSSV-free shrimp (shrimp 1 and shrimp 2) and WSSV-resistant shrimp (GenBank accession numbers GU645437∼GU645531) were compared. The WSSV-resistant shrimp were adapted to the WSSV-rich environment, with special resistance against WSSV infection [2]. Sequence comparisons revealed that some fragments of the ORF of the *PjCaspase* gene contained base mutations which were present only in the WSSV-resistant shrimp (Figs. S1, S2 and S3). This information suggested the potential involvement of these fragments in the virus-induced apoptosis against virus infection.

**Figure 2:**
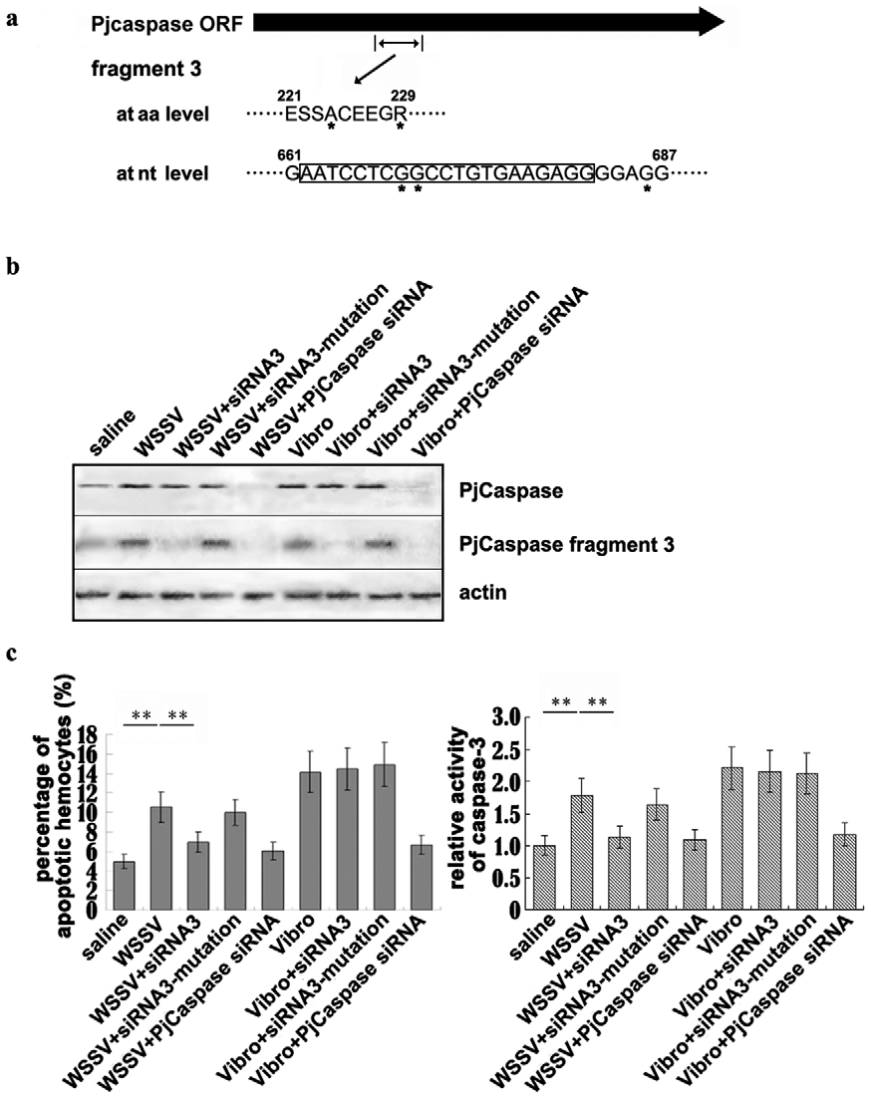
The effects on apoptosis of knocking down a *PjCaspase* gene containing fragment 3 with RNAi of shrimp hemocytes. **a.** Schematic diagram of the amino acid and nucleotide sequences of fragment 3 of the *PjCaspase* gene. The numbers showed the sites of fragment 3 in the *PjCaspase* gene sequence. The *PjCaspase* fragment 3 was obtained by comparing the *PjCaspase* gene sequence diversities of WSSV-susceptible and WSSV-resistant shrimp. The sequence targeted by fragment 3-specific siRNA (siRNA 3) was boxed. The asterisks (*) indicated where the bases or amino acids of fragment 3 differed from those of other *PjCaspase* molecules. **b.** Detections of *PjCaspase* mRNA by Northern blots with DIG-labeled gene-specific probes using the total RNAs extracted from shrimp hemolymph. The probes used were indicated on the right. The sequence of the *PjCaspase* probe (5′-CACCTGGATTAC CTCGTCCAT-3′) was identical in all *PjCaspase* molecules, while the sequence of the *PjCaspase* fragment 3 probe (5′-CCTCTTCACAGGCCGAGGATT-3′) was specific for the *PjCaspase* fragment 3 only. Shrimp*β-actin* was used as a control. Shrimp were injected with different solutions as shown in the lane headings, respectively. Subsequently, the shrimp hemocytic apoptosis was induced by WSSV or pathogenic *Vibrio parahaemolyticus*. After injection, the shrimp hemolymph was collected at 36 h and subjected to Northern blot and apoptotic assays. **c.** Evaluations of shrimp hemocytic apoptotic activities including DAPI staining of hemocytes (left) and detection of caspase 3 activity (right). The relative activity of caspase 3 was indicated by comparison with the negative control (siRNA buffer). The shrimps used in these assays were WSSV-sensitive. All the assays were biologically repeated three times. Each column indicated the mean of triplicate assays, with the standard deviation shown as a bar. Row headings represented the solutions used for injections. The asterisks (**) indicated the significant differences between treatments of interests (p<0.01).

To reveal the roles of these fragments in apoptosis against virus, the fragment-sequence-specific siRNAs were used to manipulate the expression of the *PjCaspase* gene separately containing these fragments, followed by WSSV- or *Vibrio parahaemolyticus* (a pathogen of shrimp)-induced apoptosis assays. The results showed that the *PjCaspase* gene containing a specific fragment (661–687 nt) (Fig. 2a) (fragment 3) displayed the highest apoptotic activity. At the same time, silencing the expression of the *PjCaspase* gene containing other fragments did not create any significant effect on the shrimp apoptotic activity (data not shown). Therefore the *PjCaspase* gene containing fragment 3 was further characterized.

To understand the biological function of the *PjCaspase* gene containing fragment 3, an RNAi assay was conducted by injecting the fragment 3-specific siRNA (siRNA3) into shrimp to silence the expression of the *PjCaspase* gene containing fragment 3. Interestingly, the injection completely suppressed the expression of the *PjCaspase* mRNA containing fragment 3 in the shrimps treated with the siRNA3 (Fig. 2b). On the other hand, the expression levels of the other *PjCaspase* mRNAs were not affected by the injection of siRNA3 (Fig. 2b). As control, a single nucleotide mutation was randomly introduced into the sequence of the siRNA3, resulting in a siRNA3-mutation. Surprisingly, the single random mutation abolished the siRNA3's ability to silence the expression of the *PjCaspase* mRNA containing fragment 3 (Fig. 2b). Our results indicated that the siRNA3 regulated only the *PjCaspase* mRNA containing fragment 3, and not the expression of the other genes.

Under the condition that the expression of the *PjCaspase* gene containing fragment 3 was silenced by the siRNA3, we examined the ability of the siRNA3 to affect the apoptosis induced by WSSV or *Vibrio parahaemolyticus*. As shown in Fig. 2c (lanes labeled “WSSV+PjCaspase siRNA” and “Vibro+PjCaspase siRNA”), the silencing of the *PjCaspase* gene by siRNA caused a statistically significant decrease (p<0.01) in the apoptotic activities of shrimp hemocytes induced by WSSV or *Vibrio*, showing that the PjCaspase was required in the apoptosis of virus- or bacterium-challenged shrimp.

Interestingly, the results indicated that the down-regulation by the siRNA3 of the *PjCaspase* gene containing fragment 3 led to a 1.5-times decrease (statistically significant, p<0.01) of WSSV-induced apoptotic activities of shrimp hemocytes, with the positive control, but did not influence the *Vibrio*-induced apoptosis, as compared with controls (Fig. 2c). Taken together, our results suggested that only the *PjCaspase* gene containing fragment 3 was involved in the virus-induced apoptosis, although there were diverse *PjCaspase* molecules in shrimp (*i. e.* gene sequence diversity).

**Figure 3:**
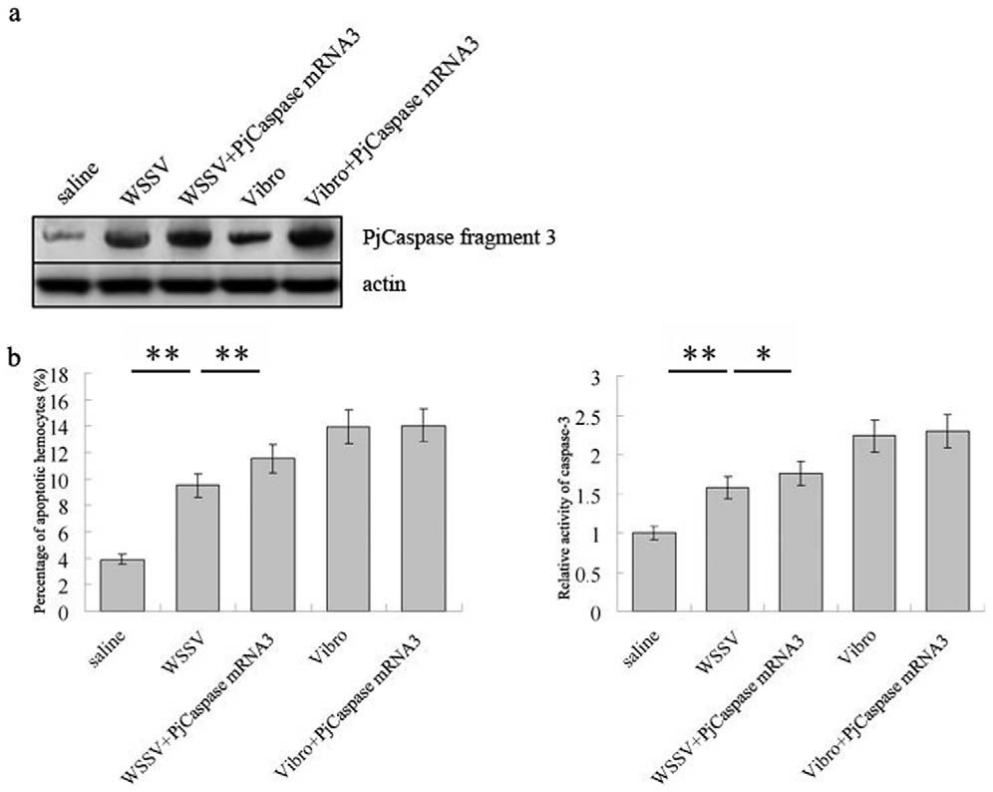
The effects of overexpression of the *PjCaspase* gene containing fragment 3 on apoptotic activities of shrimp hemocytes. The *in vitro–*synthesized mRNA of *PjCaspase* containing fragment 3 (mRNA 3) was injected into the shrimp. The shrimp apoptosis was simultaneously induced by WSSV or pathogenic *Vibrio parahaemolyticus*. After the mRNA injection, the shrimp hemolymph was collected at 36 h and subjected to Northern blot and apoptotic assays, respectively. a. Detections of *PjCaspase* mRNA containing fragment 3 (PjCaspase mRNA3) by Northern blots with DIG-labeled gene-specific probes using the total RNAs extracted from shrimp hemolymph. The probes used were indicated on the right. Shrimp *β-actin* was used as control. Row headings indicated the solutions used for injections. b. Apoptotic activities of shrimp hemocytes, including DAPI staining of hemocytes (left) and detection of caspase 3 activity (right). The relative activity of caspase 3 was indicated by comparison with the negative control (mRNA buffer). All shrimp used were WSSV- sensitive shrimp. All the assays were conducted in triplicate. Each column represented the mean of triplicate assays with standard deviation shown as a bar. Row headings showed the solutions used for injections. The asterisks (*) or double asterisks (**) indicated the significant differences between treatments of interest (p<0.05 or p<0.01, respectively).

To illustrate the effect of overexpression of the *PjCaspase* gene containing fragment 3 on apoptosis of shrimp hemocytes, the *PjCaspase* mRNA containing fragment 3 (mRNA3) was synthesized *in vitro* and injected into shrimp. The Northern blot analysis indicated that the injection increased the levels of the *PjCaspase* mRNA3 in shrimp hemocytes (Fig. 3a). In this case, the WSSV-induced apoptotic activities were significantly increased or in shrimp hemocytes with high levels of the mRNA3 by 1.2 times (p<0.01), as compared to the controls (p<0.05), whereas the *Vibrio*-induced apoptosis remained unchanged (Fig. 3b).

As a control, the mRNA of glutathione S-transferases (GST) was injected into shrimp. The results showed that the expression of the *PjCaspase* gene containing fragment 3 and shrimp apoptosis were not affected by the GST mRNA (data not shown). Overall, our results indicated that the *PjCaspase* molecule containing fragment 3 was specifically responsible for virus-induced apoptosis, and that the *PjCaspase* gene sequence diversification might contribute to the pathogen-specific immunity in shrimp.

### The role of virus-specific *PjCaspase* molecule in immunity against virus infection

The preceding data indicated that the *PjCaspase* gene containing fragment 3 was specifically responsible for the virus-induced apoptosis. How would this virus-specific *PjCaspase* molecule function in the shrimp's immunity against virus infection? To address this question, the WSSV replication in shrimp with low or high levels of the *PjCaspase* gene containing fragment 3 was monitored using quantitative real-time PCR.

**Figure 4:**
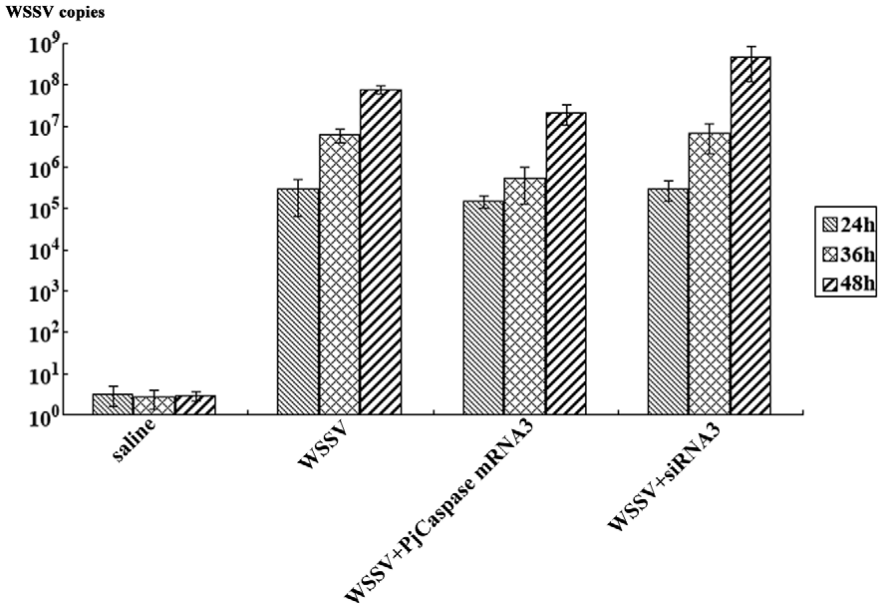
The role of *PjCaspase* gene containing fragment 3 in immunity against virus infection. The *PjCaspase* gene containing fragment 3 was respectively silenced by *PjCaspase* fragment 3-specific siRNA (siRNA 3) or overexpressed by injection of the *in vitro* synthesized *PjCaspase* fragment 3 mRNA (mRNA 3), followed by infection with WSSV. At different stages after virus infection (24 h, 36 h and 48 h), the shrimp hemolymph was collected and subjected to quantitative real-time PCR to monitor the WSSV replication. The shrimp used in the assays were WSSV-sensitive. All the assays were biologically repeated for three times. Each column indicated the mean of triplicate assays, with the standard deviation shown as a bar. Row headings represent the solutions used for injections.

The quantitative analyses revealed that when the *PjCaspase* gene containing fragment 3 was silenced by the siRNA3, the frequency of copies of WSSV in shrimp was 6 times higher than that of the control (WSSV only) at 48 h post-infection (p.i.) with WSSV (Fig. 4). On the other hand, the overexpression of the *PjCaspase* gene containing fragment 3 led to an 11-fold decrease of virus copies, compared to the control (WSSV only) at 36 h after WSSV infection (Fig. 4). These results confirmed that the *PjCaspase* gene containing fragment 3 could specifically inhibit the proliferation of WSSV.

## Discussion

Vertebrate adaptive immunity is achieved through the vast diversity of somatically rearranged immunological receptors, such as antibodies, to initiate the specific immune response against different invading pathogens [22]. Whether invertebrates are capable of a comparable phenotypic plasticity and specificity has long been a matter of debate. The results of this study on the function of gene sequence diversity in invertebrate immunity shed light on this issue.

**Figure 5:**
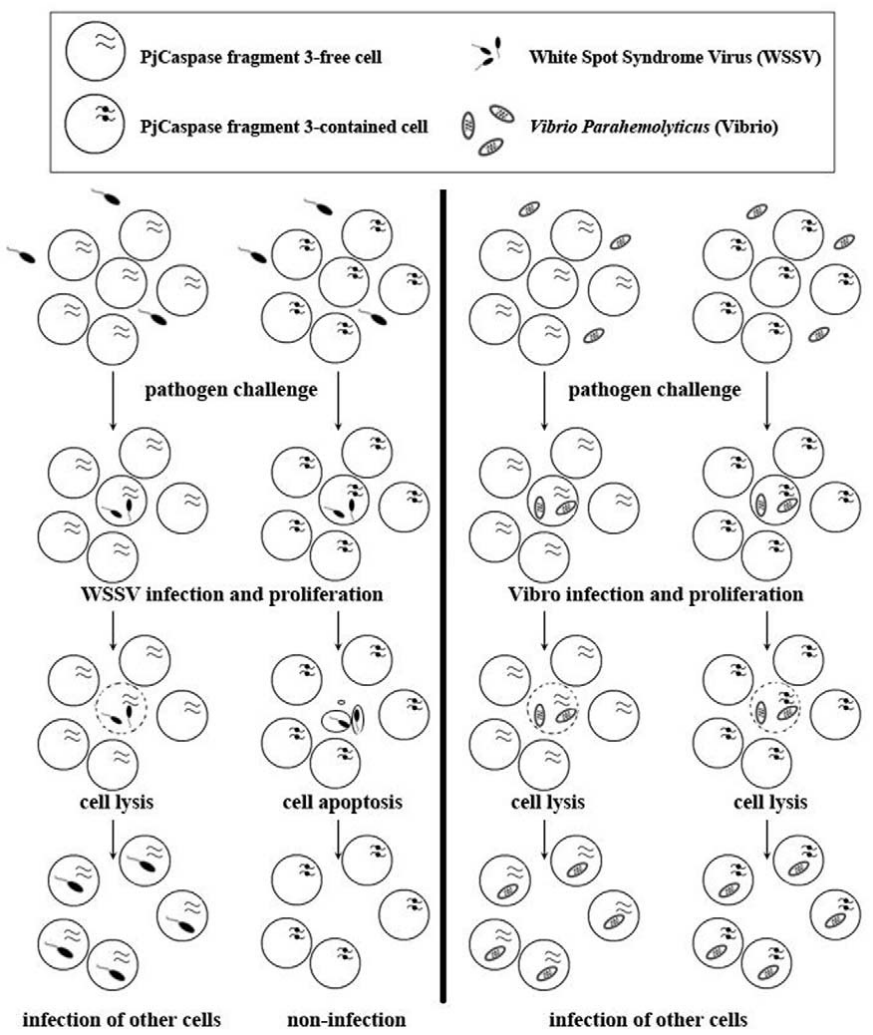
The model for the role of the *PjCaspase* gene containing fragment 3 in immunity against virus infection. When the shrimp was challenged by various pathogens, such as WSSV or *Vibrio parahaemolyticus*, the *PjCaspase* gene containing fragment 3 produced by gene sequence diversification was specifically initiated to induce apoptosis in response to virus infection. Subsequently the virus infection was inhibited by apoptosis in cells carrying the *PjCaspase* gene containing fragment 3. The apoptosis caused by *Vibrio* was not included in this model.

Based on the results derived from this study, a model for the role of *PjCaspase* gene sequence diversity in innate immunity was proposed (Fig. 5). In this model, the apoptosis caused by *Vibrio* was not included. The shrimp's pre-existing host-defense mutation mechanism produced the *PjCaspase* genes with different base mutations, resulting in the *PjCaspase* gene sequence diversity. Among the diversified *PjCaspase* genes, there was a special *PjCaspase* molecule containing fragment 3 which existed in a group of cells. When challenged by various pathogens, such as viruses and bacteria, the *PjCaspase* gene containing fragment 3 responded to the threat of a WSSV virus infection by specifically initiating the apoptosis which inhibited the WSSV infection. The PjCaspase protein, an initiator in the caspase cascade, was required as part of the apoptotic pathway of shrimp [16].

Recent studies revealed that the WSSV perturbed the function of shrimp caspase as part of its infection cycle [23], [24]. Therefore, we can infer that the sequence diversity of the *PjCaspase* gene was employed as a very efficient immune strategy to defend against various invading pathogens. In this system, the diversified *PjCaspase* molecules (gene sequence diversity) responded to different pathogens through its death signal receptor. The hypothesized mechanism might be similar to the immunoglobulin diversification in vertebrate immunity. Considering the broad existence of sequence diversity of immune-related genes in invertebrates [21, 25 and this study], gene sequence diversity might play very important roles in invertebrate immune reaction against virus infection. The gene sequence diversification in invertebrates could ensure a broad defense to viruses, bacteria and parasites. Finding this immune feature in invertebrates should prompt us to revisit the artificial dichotomy between specific and non-specific immune systems. Unusual genetic mechanisms for diversifying recognition proteins might be a widespread characteristic of animal immunity [26].

The gene sequence diversity employed by invertebrates as an efficient immune strategy against various pathogens might have developed through evolution. Darwin's theory of evolution tells us that when random genetic mutations occur within an organism, the organism preserves the beneficial mutations because they aid in survival. Among the cells of an individual invertebrate, random genetic mutations (i.e. gene sequence diversity) existed in some key genes, such as the *PjCaspase* gene revealed in this study. When various pathogens invaded the shrimp, the diversified *PjCaspase* genes initiated the apoptosis as an immune defense against virus infection. In the absence of acquired immunity, therefore, the shrimp achieved immunity against different pathogens through the vast variety of somatically diversified, pathogen-specific immunological gene sequences. The gene sequence diversity might not function as a pathogen recognition mechanism like immunoglubin, but instead as a system that avoided pathogen interference in the immune response.

Immunity studies in vertebrate and invertebrate species suggest that the invertebrate defense mechanisms may be precursors of vertebrate immunity. The diversity of gene sequences may be an ancient immune response against virus infection.

## Materials and Methods

### Shrimp culture, white spot syndrome virus (WSSV) infection of shrimp and WSSV-resistant shrimp

*Marsupenaeus japonicus* shrimps, about 10 g and 10–12 cm in length, were obtained in Xiamen, Fujian province of China and raised in groups of 20 individuals in 80-liter aquariums filled with air-pumped circulating sea water at 25°C. For the virus injection assay, the tissues of WSSV-infected shrimp (confirmed by PCR) were homogenized in TN buffer (20 mM Tris-HCl, 400 mM NaCl, pH 7.4) at 0.1 g/ml, followed by centrifugation at 2,000×g for 10 min [2]. The supernatant was diluted to 1∶100 with filter-sterilized 0.9% NaCl. Subsequently, 0.1 ml of filtrate (10^4^ virus copies/ml) was injected intramuscularly into virus- free shrimp in the lateral area of the fourth abdominal segment using a syringe with a 29-gauge needle. At different time intervals p.i., the hemolymph of three randomly selected specimens was collected, mixed and immediately stored at −80°C. In order to obtain WSSV-resistant shrimp, the virus-free shrimp was challenged with WSSV by intramuscular injections weekly over a four-week period, as described by Wu et al. [2]. The survivors (approximately 1–5%) were collected as WSSV-resistant shrimp (a pre-existing subpopulation resistant against WSSV) which were WSSV-free as detected by PCR. The unchallenged shrimp were used as a control.

### Challenge of shrimp by its pathogenic bacterium *Vibrio parahaemolyticus*

*Vibrio parahaemolyticus*, a pathogen of shrimp, was grown in LB (Luria-Bertani) medium [27]. The challenge of shrimp by *Vibrio parahaemolyticus* was conducted by intramuscular injection of 0.1 ml of bacterial suspension (1×10^6^ CFU/ml) in the lateral area of the fourth abdominal segment, using a syringe with a 29-gauge needle. At different times p.i., the hemolymph of three specimens randomly selected was collected, mixed and immediately stored at −80°C for later use.

### Cloning and sequencing of *PjCaspase* cDNA

Total RNAs of different tissues or organs including hemolymph, hepatopancreas, muscle, gill and intestine from a shrimp individual were extracted using TRIzol kit according to the manufacturer's instructions (Invitrogen, Carlsbad, CA, USA), followed by treatment with DNase I (Roche, Grenzacherstrasse, Basel, Switzerland) to remove any genomic DNA contamination. The concentration of total RNAs was determined spectrophotometrically at OD_260_ and the integrity of total RNAs was examined by electrophoresis on a 2% agarose gel. The extracted RNAs were stored at ^_^80°C for future use.

For reverse transcription, 1 µg of total RNAs was mixed with 1 µl oligo-d(T)18 primer and diethypyrocarbonate (DEPC)-treated water to a final volume of 12 µl. After incubation at 70°C for 5 min, the mixture was cooled on ice. A reaction mix of 1 µl M-MLV RT (Promega, Madison, WI, USA), 1 µl dNTP (10 mM each; Promega), 1 µl recombinant RNasin ribonuclease inhibitor (Promega), 5 µl M-MLV 5 × reaction buffer (Promega) and 5 µl DEPC-treated water were mixed and incubated at 42°C for 60 min. The reaction was terminated by heating at 70°C for 15 min. The resulting cDNAs were stored at ^_^20°C until further use.

The *PjCaspase* cDNA was amplified using two *PjCaspase* gene-specific primers (5′-ATGGACGAGGTAATCCAG-3′ and 5′-TCACTGTGGGGCGGAG-3′) [16]. As a control, the shrimp *β-actin* gene was included in the amplification with primers 5′-ATGTGT GACGACGAAGTAGC-3′ and 5′-GAAGCACTTCCTGTGAACGA-3′. PCR was conducted with an initial denaturation step of 5 min at 94°C, followed by 30 cycles of 30 s at 94°C, 30 s at 50°C and 60 s at 72°C. All the PCRs were performed with Pfx DNA polymerase (Invitrogen, Carlsbad, CA, USA). PCR products were purified (Omega Bio-tek, Norcross, GA, USA) and cloned into pMD18-T vector. Twenty of the *PjCaspase* cDNA clones on a plate were selected randomly for sequencing.

### Statistical analysis of sequences

To infer the diversification of variations in the *PjCaspase* cDNA sequences, as well as in the *actin* cDNA sequences, the criterion reported by Zhang *et al*. [21] was used to draw out the reliable sequences as the sources which were considered as original and un-divergent. The total variation number in each cluster was minimized.

### Southern blot analysis

Genomic DNA was prepared from shrimp hemolymph using a SQ tissue DNA Kit (Omega Bio-tek, Norcross, GA, USA) according to the manufacturer's instructions. Briefly, the fresh shrimp hemolymph was homogenized thoroughly in the WTL buffer using a microfuge tube pestle. After adding proteinase K solution (20 mg/ml), the homogenized mixture was incubated at 60°C for 3 hours. Thereafter, RNase A solution was added to the mixture and incubated for 15 min at 37°C. The PCP buffer was added and vortexed vigorously for 30 seconds, followed by incubation on ice for 5 min and centrifugation at 13,000 g for 3 min at room temperature. Subsequently the supernatant was mixed with 100% isopropanol and centrifuged at 14,000 g for 1 min at room temperature. After washing with 70% ethanol several times, the genomic DNA was resuspended in sterile water and stored at 2°–8°C for later use.

The genomic DNA extracted from shrimp hemolymph was digested with DraI or EcoRI, respectively, and then separated by electrophoresis on a 0.7% agarose gel in 0.5×TBE buffer (90 mM Tris-boric acid, 2 mM EDTA, PH 8.0). The separated DNA was transferred to a nylon membrane (Amersham Biosciences, Fairfield, CT, USA). The blots were probed with a DIG-labeled *PjCaspase* gene that could detect all mutations of *PjCaspase*. For the labeling of *PjCaspase* gene, the full-length gene was amplified by PCR and randomly labeled with DIG. The DIG labeling and detection were performed following the protocol of the DIG High Prime DNA Labeling and Detection Starter Kit (Roche, Grenzacherstrasse, Basel, Switzerland).

### Synthesis of siRNA

The siRNAs used in this study consisted of 21-nucleotide double-stranded RNAs, each strand of which contained a 17∼22-nucleotide target sequence and a two-uracil (U) overhang at the 3′ end [28]. Based on the different sequences between the caspases from WSSV- resistant shrimp and virus-free shrimp, 6 fragments of the *PjCaspase* gene being prognosticated to be involved in shrimp antiviral defense were selected for RNAi assays. The siRNAs were designed according to the sequences of 6 fragments of the *PjCaspase* gene. The sequences of siRNAs respectively targeting the 6 fragments of *PjCaspase* gene were as follows:

fragment 15′-TTGGATCCTACCGTAGGAGTG-3′

fragment 25′-T TGAGGTACTCCTGCAGGCT T-3′

fragment 35′-CCTCTTCACAGGCCGAGG ATT-3′

fragment 45′-ATAGAACT GGTATTCAGGCTT-3′

fragment 55′-TTAGCACCTTCAAGTCTGAATT-3′

fragment 65′-CGAAAGGTGGTCAGG AATGGTT-3′

The siRNA sequence of fragment 3 (siRNA3), showing the highest apoptotic activity, was randomly mutated at one nucleotide, resulting in siRNA 3-mutation (5′-CCTATTCACAGGCCGAGGATT-3′). The sequence of siRNA having RNAi capacity to all *PjCaspase* mRNA molecules was 5′-GTTATGAAGTGCTACAG-3′ (PjCaspase siRNA).

The siRNAs were synthesized in vitro, using a commercially available kit, according to the manufacturer's recommendations (TaKaRa, Japan). The formation of double-stranded RNAs was monitored by determining the size shift in agarose gel electrophoresis. The synthesized siRNAs were dissolved in siRNA buffer (50 mM Tris-HCl, 100 mM NaCl, PH 7.5) and quantified spectrophotometrically.

### RNAi assay of shrimp in vivo

The RNAi assays were conducted with the following 9 treatments: physiological saline only; WSSV (10^5^ virion copies/ml) only; WSSV (10^5^ virion copies/ml)+siRNA 3 (24 µM); WSSV (10^5^ virion copies/ml)+siRNA 3-mutation (24 µM); WSSV (10^5^ virion copies/ml)+ PjCaspase siRNA (24 µM); *Vibrio parahaemolyticus* (10^6^ copies/ml) only; *Vibrio parahaemolyticus* (10^6^ copies/ml)+siRNA 3; *Vibrio parahaemolyticus* (10^6^ copies/ml)+siRNA 3-mutation and *Vibrio parahaemolyticus* (10^6^ copies/ml)+ PjCaspase siRNA).

The WSSV, *Vibrio parahaemolyticus* and/or siRNA dissolved in physiological saline solution were delivered into the shrimp by simultaneous injection into the juncture area between the third and forth abdominal section at 0.1 ml/shrimp, using a syringe with a 29-gauge needle. At different time intervals p.i., the shrimp hemolymph was collected from the ventral sinus by inserting a 26-gauge needle containing heparin sodium (500 U/ml). Three shrimp specimens from each treatment, selected at random, were subjected to Northern blot and apoptosis assays, including detection of caspase-3 activity and DAPI staining of shrimp hemocytes. All assays were conducted in biological triplicate.

### Synthesis of *PjCaspase* mRNA containing fragment 3 and its over-expression in shrimp

The synthesis of *PjCaspase* mRNA containing fragment 3 and its over-expression in shrimp were conducted as described previously (Wu et al., 2008). To synthesize the *PjCaspase* mRNA containing fragment 3, the *PjCaspase* gene was divided into two segments, segment 1 (from the 5′ terminal of the *PjCaspase* gene to fragment 3) and segment 2 (from fragment 3 to the 3′ terminal of the *PjCaspase* gene). Segment 1 was amplified by PCR with primers 5′-GATCA CTAATACGACTCACTATAGGGATGGACGAGGTAATCCAGGT-3′ (T7 promoter, underlined) and 5′-CCTCTTCACAGGCCGAGGATT-3′ (sequence of fragment 3). Segment 2 was amplified by PCR with primers 5′-AATCCTCGGCCTGTGAAGAGG-3′ (complementary sequence of fragment 3) and 5′-TCACTGTGGGGCGGAGTGGA-3′. After amplifications, segments 1 and 2 were denatured at 99°C and annealed. The annealed DNAs were amplified by PCR with primers 5′-GATCACTAATACGACTCACTATAGG-3′ and 5′-TCACTGTGGGGCGGAGTGGA-3′ to screen for the fragment-3-containing templates in which the sequence of fragment 3 from segment 1 was paired with the complementary sequence of fragment 3 from segment 2. Subsequently the capped *PjCaspase* mRNA containing fragment 3 was transcribed from the fragment-3-containing templates with a T7 RNA polymerase *in vitro* transcription kit (TaKaRa Bio, Dalian, Liaoning, China) according to the manufacturer's instructions.

The synthesized *PjCaspase* mRNA containing fragment 3 (20 µg/shrimp) was delivered into the shrimp at the juncture area between the third and forth abdominal section at 0.1 ml/shrimp using a syringe with a 29-gauge needle. Twelve hours later, the *PjCaspase* mRNA containing fragment 3 (20 µg/shrimp) was simultaneously injected into shrimp with WSSV (10^5^ virion copies/ml) or *Vibrio parahaemolyticus* (10^6^ copies/ml), respectively. As controls, the physiological saline only, WSSV (10^5^ virion copies/ml) only and *Vibrio parahaemolyticus* (10^6^ copies/ml) only were included in the injections. At various times after injection, the shrimp hemolymph was collected from the ventral sinus by inserting a 26-gauge needle containing heparin sodium (500 U/ml). Three shrimp specimens from each treatment, selected at random, were subjected to Northern blot and apoptosis assays, including detection of caspase-3 activity and DAPI staining of shrimp hemocytes. All assays were biologically repeated three times.

### Northern blot

Total RNAs were extracted from shrimp hemolymph using TRIzol reagent kit (Invitrogen, USA). After separation in a 1.2% agrose gel, total RNAs were transferred to nylon membrane (Amersham Biosciences, USA), followed by probing with DIG-labeled *PjCaspase* or shrimp *β-actin* genes. The sequence of the *PjCaspase* probe (5′-CACCTGGATT ACCTCGTCCAT-3′) was identical in all *PjCaspase* molecules, while the sequence of the *PjCaspase* fragment 3 probe (5′-CCTCTTCACAGGCCGAGGATT-3′) was specific for *PjCaspase* fragment 3 only. DIG labeling and detection were conducted using DIG High Prime DNA Labeling and Detection Starter Kit II (Roche, Grenzacherstrasse, Basel, Switzerland) and BCIP/NBT (Amersham Biosciences, USA).

### Detection of caspase-3 activity

As a classical measurement for evaluation of apoptosis, the shrimp caspse-3 activity was assayed. After washing with cold phosphate-buffered saline (PBS), shrimp hemolymph was disrupted in a chilled lysis buffer (25 mM HEPES, 5 mM MgCl2, 5 mM EDTA, 5 mM dithiothreitol, 2 mM phenylmethylsulphonyl fluoride, pH 7.5). The lysate was centrifuged at 13,000×g for 5 min. The protein concentration of the supernatant was determined using Bradford Protein Assay Kit (Bio-Rad Laboratories, Hercules, CA, USA) and adjusted to 0.02 µg protein/µl. Subsequently, its caspase-3 activity was detected using the Caspase-3 Colorrimetric Assay Kit (Keygen Biotech. Co., Ltd, Nanjing, Jiangshu, China), as described in the manufacturer's manual. Briefly, 50 µl protein solution, 50 µl 2×reaction buffer (40 mmol/L PIPES, 200 mmol/L NaCl, 2 mmol/L EDTA, 0.2% CHAPS, 20% sucrose, 20 mmol/L DTT, pH 7.2) and 5 µl caspase-3 substrate were mixed and then incubated at 37°C for 4 hours. The enzyme activity of caspase-3 was measured by monitoring the absorbance at 405 nm using a Perkin-Elmer LS50B fluorimeter. All assays were performed in triplicate.

### DAPI staining of shrimp hemocytes

Shrimp hemocytes were smeared onto poly-L-lysine–treated slides and air-dried. Then the hemocytes were fixed by paraformaldehyde solution (4% in PBS) for 30 min. After rinsing with 4, 6 diamidine-2-phenyllindole dihydrochloride (DAPI) solution, the slide was incubated in DAPI solution (1 µg/ml) at 37°C for 15 min. Subsequently the slide was washed with methanol before examining it using a fluorescence microscope. For each sample, 500 hemocytes were counted and scored for condensed and fragmented nuclei. The initial data was produced by NIS-Elements BR (NIKON fluorescence microscope software).

### Real-time PCR

Virion copies of WSSV were quantified using WSSV-specific primers (5′-TTGG TTTCA TGCCCGAGATT-3′ and 5′-CCTTGGTCAGCCCCTTGA-3′) and TaqMan fluorogenic probe (5′-FAM-TGCTGCCGTCTCCAA-TAMRA-3′). The viral genomic DNA was prepared from WSSV-infected shrimp using SQ Tissue DNA kit (OMEGA, America) and subjected to PCR. The PCR reaction mixture (25 µl) included WSSV genomic DNA, 200 nM of each primer, 100 nM of TaqMan probe and 1×PCR reaction buffer containing DNA polymerase. PCR amplification was performed for 4 min at 50°C, followed by 45 cycles of 30 s at 94°C, 30 s at 52°C and 30 s at 72°C.

### Statistical analysis

All numerical data from biologically independent experiments were analyzed using a one-way analysis of variance (ANOVA) to calculate the mean and standard deviation of repeated assays. Comparison of differences among treatments was carried out using Student's *t* test.

## Supporting Information

Figure S1 **The sequence alignments of PjCaspase cDNA from various shrimp organs or tissues (shrimp 1).** The shrimp *actin* gene was used as control. The numbers on the right showed the positions of nucleotide or amino acid, respectively. All point mutations were indicated below the original sequences. The mutations at the same position were shown with numbers on the top. **a.** The nucleotide sequence alignment and mutation analysis of the *PjCaspase* gene. **b.** The amino acid sequence alignment and mutation analysis of the *PjCaspase* gene. **c.** The nucleotide sequence alignment and mutation analysis of the shrimp *actin* gene. **d.** The amino acid sequence alignment and mutation analysis of shrimp *actin*.
(TIF)

Figure S2 **The sequence alignments of** ***PjCaspase*** **cDNA from various shrimp organs or tissues (shrimp 2).** The numbers on the right showed the positions of nucleotide or amino acid, respectively. All point mutations were indicated below the original sequences. The mutations at the same position were shown with numbers on the top. **a.** The nucleotide sequence alignment and mutation analysis of the *PjCaspase* gene. **b.** The amino acid sequence alignment and mutation analysis of the *PjCaspase* gene.
(TIF)

Figure S3 **The genomic DNA sequence alignments of the shrimp** ***PjCaspase*** **gene.** The shrimp *actin* gene was used as control. The numbers on the right showed the positions of nucleotide. All point mutations were indicated below the original sequences. The mutations at the same position were shown with numbers on the top. **a.** The genomic DNA sequence alignment and mutation analysis of the *PjCaspase* gene. **b.** The genomic DNA sequence alignment and mutation analysis of the *actin* gene.
(TIF)
