# Supplementary figures and images for: Contribution of the Caspase Gene Sequence Diversification to the Specifically Antiviral Defense in Invertebrate
Source: PLoS One. 2011 Sep 19;6(9):e24955. doi: 10.1371/journal.pone.0024955 (PMC3176291; doi:10.1371/journal.pone.0024955)

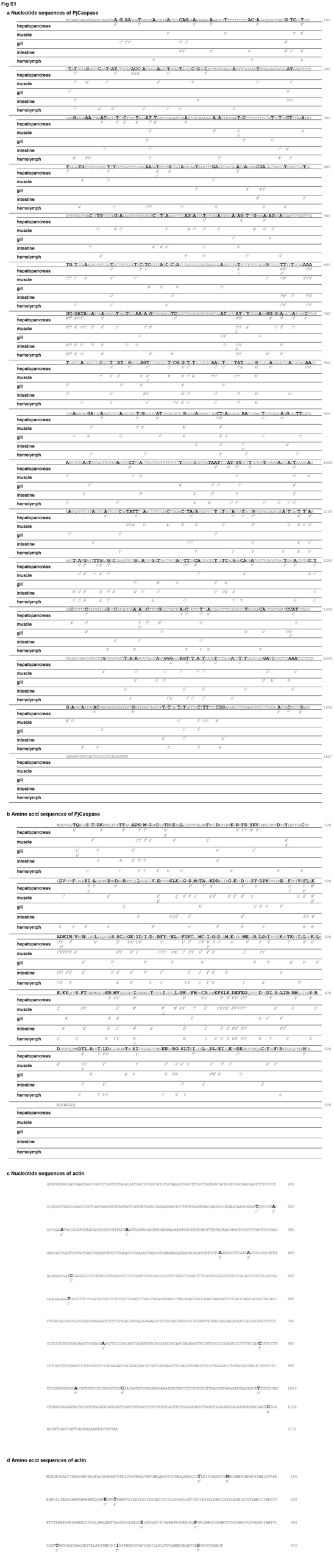

Supplement: Figure S1 — The sequence alignments of PjCaspase cDNA from various shrimp organs or tissues (shrimp 1). The shrimp actin gene was used as control. The numbers on the right showed the positions of nucleotide or amino acid, respectively. All point mutations were indicated below the original sequences. The mutations at the same position were shown with numbers on the top. a. The nucleotide sequence alignment and mutation analysis of the PjCaspase gene. b. The amino acid sequence alignment and mutation analysis of the PjCaspase gene. c. The nucleotide sequence alignment and mutation analysis of the shrimp actin gene. d. The amino acid sequence alignment and mutation analysis of shrimp actin. (TIF) [file pone.0024955.s001.tif]

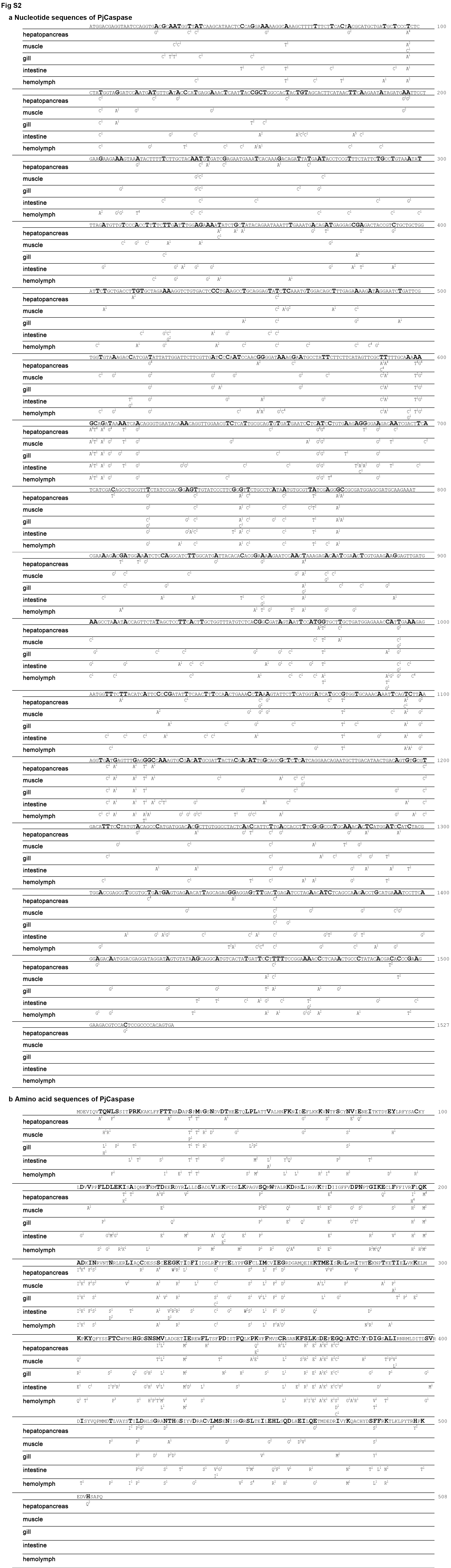

Supplement: Figure S2 — The sequence alignments of PjCaspase cDNA from various shrimp organs or tissues (shrimp 2). The numbers on the right showed the positions of nucleotide or amino acid, respectively. All point mutations were indicated below the original sequences. The mutations at the same position were shown with numbers on the top. a. The nucleotide sequence alignment and mutation analysis of the PjCaspase gene. b. The amino acid sequence alignment and mutation analysis of the PjCaspase gene. (TIF) [file pone.0024955.s002.tif]

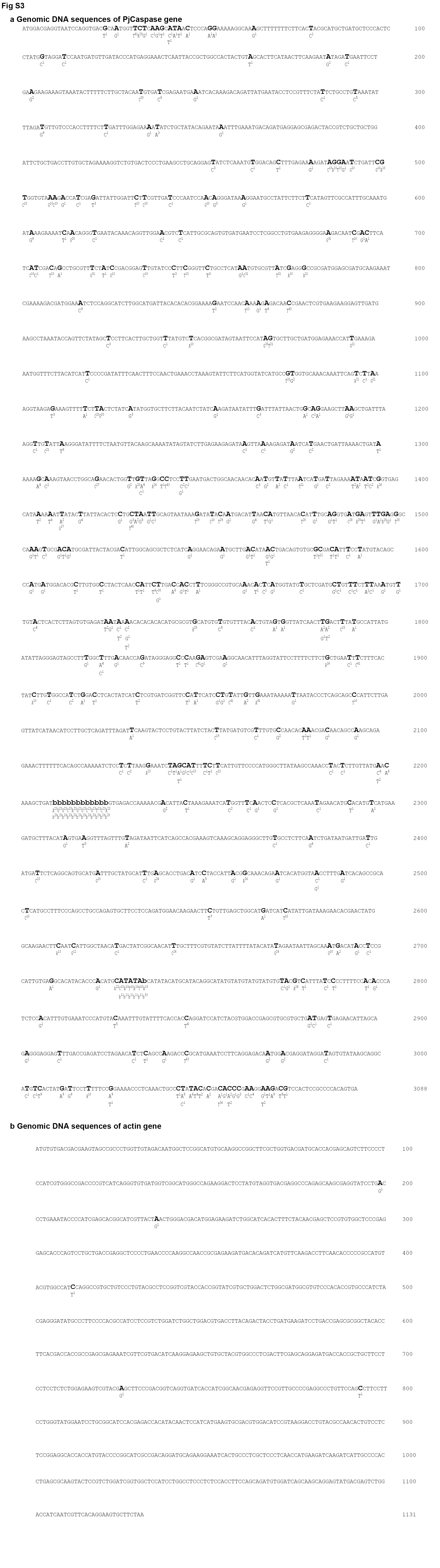

Supplement: Figure S3 — The genomic DNA sequence alignments of the shrimp PjCaspase gene. The shrimp actin gene was used as control. The numbers on the right showed the positions of nucleotide. All point mutations were indicated below the original sequences. The mutations at the same position were shown with numbers on the top. a. The genomic DNA sequence alignment and mutation analysis of the PjCaspase gene. b. The genomic DNA sequence alignment and mutation analysis of the actin gene. (TIF) [file pone.0024955.s003.tif]
